# An Analysis of Waiting Times for the Diagnosis and Treatment of Patients with Prostate Cancer Established by the Requirements of the Fast-Track Cancer Treatment Pathway, Taking into Account Treatment Steps

**DOI:** 10.3390/cancers17111842

**Published:** 2025-05-31

**Authors:** Aleksandra Sierocka, Stanisław Brzozowski, Michał Marczak, Mariusz Bednarek, Remigiusz Kozłowski

**Affiliations:** 1 Department of Management and Logistics in Healthcare, Medical University of Lodz, 90-419 Lodz, Poland; remigiusz.kozlowski@umed.lodz.pl; 2 Department of Management, SAN University in Lodz, 90-113 Lodz, Poland; stan.brzozowski@gmail.com; 3 Department of Innovation, Merito University in Poznan, 61-895 Poznan, Poland; michal.j.marczak@gmail.com (M.M.); mariusz.bednarek@wsb.warszawa.pl (M.B.); 4 Campus Santiago, Universidad Autonoma de Chile, Temuco 4810101, Chile

**Keywords:** prostate cancer, fast-track cancer treatment pathway, cost-intensity of treatment, visualization of treatment processes, benefit value flow diagram, analysis of diagnosis and treatment times, Lean Healthcare 4.0 diagnostic model

## Abstract

Prostate cancer is the most common cancer in men in 112 countries, accounting for 1 in 14 diagnosed cancer cases worldwide. For this reason, the diagnosis and treatment of prostate cancer changed significantly, becoming a multidisciplinary process. Since 2015 in Poland, measures have been taken to accelerate the diagnosis and treatment of cancers, including shortening patients’ diagnosis and treatment waiting times. Background/Objectives: The aim of this study was to characterize the current clinical pathway of patients with prostate cancer and to establish standard times for various steps of treatment, as well as to develop a visualization of the steps involved in diagnosis and treatment. Methods: The research covers 2018 to 2022 in a large oncological hospital in Poland (EU area). The source data, in the form of 10 fully anonymized CSV files generated from the hospital’s medical statistics, have been converted to the target data model. Results: The time limits for diagnosing and commencing the treatment of patients with diagnosed prostate cancer specified by legal regulations and by guidelines of scientific associations are not met in 42% of cases. The greatest delays concern the initiation of the steps of treatment (53%) and comprehensive diagnostics (37%). The diagnostic pathways should be modified to facilitate early and rapid detection of prostate cancer and to allow further therapy within the time limit strictly defined by regulations and guidelines of scientific associations.

## Simple Summary

Prostate cancer is the most common cancer in men globally, prompting major changes in its diagnosis and treatment, which are now multidisciplinary. In Poland, efforts since 2015 aimed to shorten waiting times for cancer care. This study analyzed the clinical pathway of prostate cancer patients in a large Polish oncology hospital between 2018 and 2022. Results show that in 42% of cases, legal and guideline-defined timeframes for diagnosis and treatment were not met, with the biggest delays in starting treatment (53%) and completing diagnostics (37%). The study suggests revising diagnostic pathways to ensure timely detection and treatment.

## 1. Introduction

Prostate cancer (also called prostate gland cancer) is the most common cancer in terms of incidence in men in 112 countries and constitutes 1 in 14 cancers diagnosed worldwide and 15% of all male cancers. World publications indicate that this disease ranks second after lung cancer in terms of cancer mortality among men [1,2,3,4]. It presents a colossal and growing medical, societal, and, unfortunately, economic problem. Factors conducive to its formation include improper diet, tobacco smoking, genetic predispositions, and age [5]. According to registry data, in thirty years, the number of cases increased about five times. Here, we talk about the challenge of the prostate cancer epidemic. Fortunately, due to the progress in diagnostics based on the widespread determination of prostate-specific antigen (PSA) levels, the increase in mortality due to this cancer was stopped in the 1990s [6,7]. The growing awareness of patients and their families, and currently, the reimbursement of the PSA test by the Polish National Health Fund, is also important. Most cases of prostate cancer occur in the seventh and eighth decades of life [1]. Although the risk of falling ill reaches a maximum after 75 years of age, much younger patients (50 and 60 years old) may potentially benefit most from early diagnosis and radical treatment if the disease has been diagnosed sufficiently early [3,8].

The available scientific publications also indicate that the number of new cases per year will increase from 1.4 million in 2020 to 2.9 million in 2040 [9]. This increase in the number of cases cannot be prevented solely by lifestyle changes or interventions in the field of public health, and politicians and rulers must prepare strategies to deal with this.

In recent years, the approach to the diagnosis and treatment of prostate cancer has completely changed and is now multidisciplinary (multimodal). This means that from the moment of a diagnosis, doctors of various specialities are involved in the treatment of patients: urologists, radiologists, radiotherapists, clinical oncologists, histopathologists, rehabilitation specialists, sexologists, palliative care specialists, geriatricians, psychiatrists, clinical psychologists, or nuclear physicists involved in the planning of radiotherapy [10]. Other persons who provide patients with additional support at every step of treatment, from the very beginning of the diagnostic and therapeutic process, include coordinating nurses, coordinators, medical secretaries, the patient rights’ ombudsman, social workers, and data managers. The best cancer care also requires, in addition to a staff of skilled workers and financial expenditure, appropriate infrastructure and organization. A key role in the treatment of patients is played by the systematic and chronologically planned cooperation of all persons directly or indirectly involved in the treatment process. To this end, centers for the multi-specialistic treatment of cancers are being created all over the world (mainly in Western Europe), targeted at a specific organ/tumor, including centers specializing in the treatment of prostate cancer (i.e., prostate cancer units). In Poland, there are already breast cancer units, and work is underway on the launch of Lung Cancer Units. A prostate cancer unit is a center intended to provide coordinated and interdisciplinary care for patients with prostate cancer, where a specific synergy of activities occurs, including coordinated care, which ultimately results in better treatment outcomes for the patient. Numerous studies confirm that this direction is well-assessed and preferred both by patients with prostate cancer and by doctors.

In 2015, the “fast-track cancer treatment pathway” (also called the DILO oncology package) [11] was implemented in Poland. Its main objective was to streamline and accelerate the diagnosis and treatment of cancer diseases by shortening the patients’ waiting times for diagnosis and initiation of treatment and by establishing a multidisciplinary therapeutic team. This was to increase their chances of effective therapy and improve their quality of life. The introduction of comprehensive cancer care has clearly improved the situation of patients in Poland [12]. However, the changes introduced to the health system have not ended there. The next step was the publication in March 2024 of the long-awaited Act on the National Oncological Network (KSO) [13], the provisions of which are to enter into force on 1st of April 2025. Its main objective is to improve the quality and availability of oncological treatment throughout the country by creating a network of medical entities covering different levels of care (from specialist cancer centers to local medical facilities). The KSO is also intended to ensure a uniform way of dealing with a patient with a given cancer (“treatment pathways”), which guarantees access to the most current treatments, in accordance with the applicable guidelines of scientific associations. This will allow better coordination and ensure continuity of care, thereby improving the outcomes of treatment and the measures of health. It will also enable the comparison of clinical effects achieved by various medical facilities.

Despite the actions taken, the statistics on the treatment of prostate cancer in our country still, unfortunately, compare negatively to the results achieved in the countries of Western Europe. It is worth noting, however, that the prognosis itself in prostate cancer is generally quite good. According to current data, the 5-year survival rate of patients with prostate cancer is about 67% in Poland and 83% in European Union countries [14,15]. The prognosis for prostate cancer depends on many factors, starting with the degree of progression of the disease and the stage at which it was diagnosed, the effectiveness and proper selection of the therapy used, as well as the age and general condition of the patient. Prognosis in prostate cancer is related to the Gleason score [16]. In patients with a low score (2–4), the prognosis of prostate gland cancer is good. A Gleason score of seven or more is an adverse prognostic factor. A score of five or six is considered to be intermediate. As emphasized by experts, the planning of treatment and determination of prognosis in prostate cancer has to be based on the determination of progression of the disease. It is assumed that there are three groups of patients with different prognoses: cancer limited to the prostate gland, prostate cancer limited to the pelvis, and metastatic cancer, the worst prognosis.

The authors of this study share the view that many negative health consequences can be avoided through better planning and implementation of potential solutions, which include changes in diagnostic and therapeutic pathways. It is, therefore, very important to learn the actual treatment pathway for a patient with malignant prostate cancer and to verify its compliance with the recommendations of scientific associations and current medical knowledge [17]. The following objectives of the article have been formulated:

1. Determining the characteristics of the current clinical path of patients with prostate cancer undergoing treatment, with the establishment of time standards for the initiation of individual steps.
2. Developing a graphical visualization of diagnosis and treatment steps for patients within the framework of the “fast-track cancer treatment pathway”.
3. Establishing the most important indicators/analytical measures for patients with a diagnosis of C61.
4. Identifying the cost-intensiveness of the various steps of treatment.
5. Identifying the number of patients who, treated by different methods, do not meet the required waiting time criteria set by the requirements of the fast-track cancer treatment pathway, taking into account the steps of treatment.

All the specific criteria and conditions for the diagnosis and treatment of the patient (including waiting times for individual steps) were indicated by the Ministry of Health as obligatory. The authors of the study suspect that many patients are not treated pursuant to the assumptions and rules specified above, which may negatively affect their health and further prognosis while also generating enormous costs for the healthcare system. Checking our thesis and confirming or disproving it will allow for preparation for entry into the force of the KSO National Oncological Network [13], within the framework of which appropriate measures and indicators will be defined, which will have to be achieved in order to be able to continue treating cancer patients. These parameters will also be the basis for determining the level of public funding of healthcare facilities. Centers which do not comply with the requirements of the KSO Act will either cease to be financed in this respect at all, or their income will be reduced in proportion to only the number of indicators that were met (hence, the need to also concentrate on the most costly steps). The authors of the study assume that the knowledge gained during the analysis of such a large dataset will be a starting point for identifying the causes of possible problems and irregularities and proposing the implementation of specific corrective actions. This publication will, therefore, be the beginning of a series of a further 2–3 articles on the issues of state-of-the-art diagnostics and treatment of prostate cancer patients and the costs of treatment for the healthcare system. It will be based on Lean Healthcare 4.0. This name is used for the latest achievements in computer science and technology, which are now providing a quantum leap in medicine. Improvements in Lean Healthcare projects should be understood as planned changes in the organization of work in order to improve the quality and effectiveness of the treatment of patients in the chosen diagnostic and clinical process. In the combination of both of these management methods, Lean Healthcare 4.0 provides a chance to achieve a competitive advantage for the medical entity that implements it effectively.

State-of-the-art methods of process management in oncological facilities include advanced strategies and technologies used to improve the coordination and personalization of the diagnosis, treatment, and rehabilitation of patients, as well as the prevention of the recurrence of cancer. Their implementation must be preceded by an appropriate analysis.

## 2. Materials and Methods

**Figure 1:**
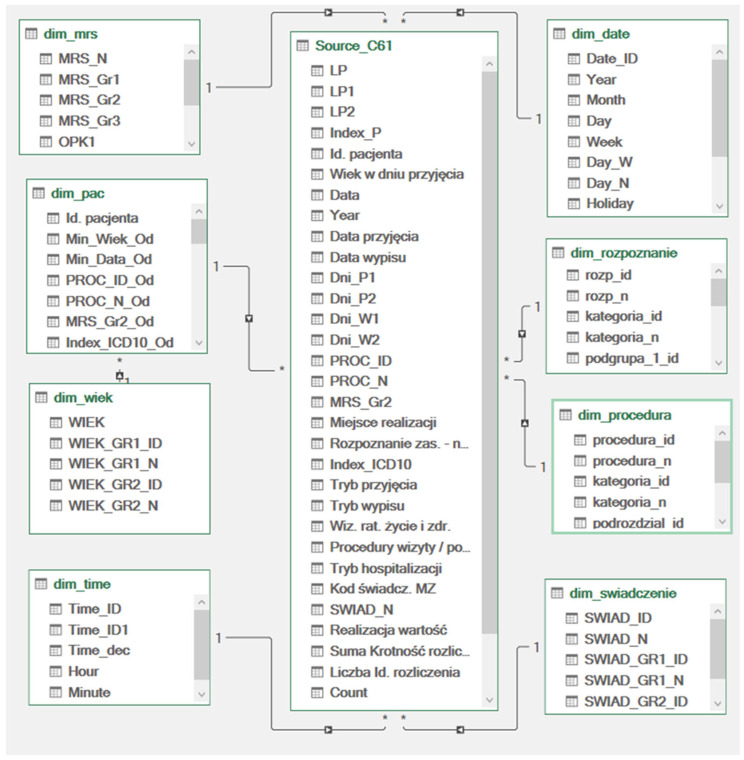
Target model of data for hospital patients with a diagnosis of C61 (own work based on the transformation of the source data received from the hospital). The model shows the links between the central medical fact table and multiple denormalized dimension tables according to the star schema in the data warehouse. The arrow symbol indicates a one-to-many relationship between the tables.

The research covers 2018 to 2022 in a large oncological hospital in Poland (EU area). The source data in the form of 10 fully anonymized CSV files generated from the hospital’s medical statistics have been converted to the target data model, the diagram of which can be found in Figure 1. The data include detailed information on individual medical events: date, place, organizational unit, patient (unique number assigned to the patient), main and co-existing diagnosis, type of services provided, manner of admission and discharge, multiple appointments, performed medical procedures, settlement items according to the NFZ National Health Fund, and price and value of settlement according to regulations of the NFZ’s President.

The representativeness of the study was achieved by defining a broad selection criterion for patients who were included in the analysis, i.e., a diagnosis of prostate cancer (C61 in the ICD-10 international classification of diseases) as the main or co-existing diagnosis. At this step, no other exclusion criteria were used, which means a large diversification of patients due to their age, severity of the disease, presence of comorbidities, methods of diagnosis, and treatment used, as well as completeness of the therapeutic process. The goal was to capture a full statistical cross-section of the population of patients of the selected hospital. Such an approach provides an opportunity to compare study results to other hospitals offering comprehensive diagnosis and treatment of patients with prostate cancer.

A single appointment was defined as the sum of all medical procedures carried out for one patient at one service provision site (SPS, e.g., a diagnostic office, hospital ward, or radiotherapy department) during the period between the date of admission and the date of discharge. If the patient received multiple medical procedures on the same day at one SPS, this means one appointment. If the patient received medical procedures on the same day in two different SPSs, this would mean two appointments, with the second appointment having zero waiting time. The key parameters calculated for each appointment are the patient’s length of stay and the patient’s waiting time (in days).

The value of the services provided is recorded in the database in PLN and converted to EUR at the exchange rate of the European Central Bank on 31 December 2024, which amounted to EUR 1 = PLN 4.275 [18].

After the addition of dictionary tables, the data model was uploaded to a MariaDB Columnstore database (version MCOL-5630) [19], which enables the execution of complex queries on large datasets in a time much shorter than for relational databases [20]. The data warehouse developed in this way was connected using the MariaDB ODBC (version 3.1) [21] mechanism with data analytics tools Power BI (version 2.128.751.0) and DAX Studio (version 3.2.1) [22]. The final versions of the resulting tables were generated using the Power Pivot add-in for Excel 365.

The result of the analysis of the data was the calculation of the most important indicators/analytical measures and the development of visualization of processes in the form of diagrams generated using the SankeyMATIC tool [23,24] for individual steps of diagnosis and treatment and the entire clinical pathway of selected patients of the oncological hospital (door-to-door).

## 3. Results

### 3.1. Characteristics of the Analyzed Process

The main assumptions of the oncological package (“fast-track cancer treatment pathway”) introduced by the Act of 22 July 2014 amending the Act on health care services financed by public funds (effective as of 1 January 2015) were as follows [11,13]:

- An oncological diagnostics and treatment card (DILO card): A document which entitles the patient to services provided under the terms set out in the package; the card may be issued by primary care physicians, ambulatory specialist care, and hospitals (at any step).
- Strictly defined maximum time for cancer diagnosis: Up to 7 weeks (including initial diagnostics within 28 days and comprehensive diagnostics within 21 days).
- Comprehensiveness and coordination of medical services: Following the completion of cancer diagnosis at a given facility, the patient remains in treatment with the same service provider or is referred to another facility for treatment, according to a plan established by a medical case conference.
- A multi-specialist therapeutic team (medical case conference), which develops an individual treatment plan optimized for the patient in accordance with current medical knowledge and treatment recommendations.
- Strictly defined maximum time for initiating treatment: Within 14 days from the date of the establishment of the treatment plan,
- Coordinator: The facility conducting the treatment assigns the patient a coordinator whose tasks include, among others, practical assistance in the implementation of the established treatment path.
- The unlimited nature of fast-track cancer treatment pathway services.
- Supervision (follow-up) of the patient after completing oncological treatment for a period of 5 years.

**Figure 2:**
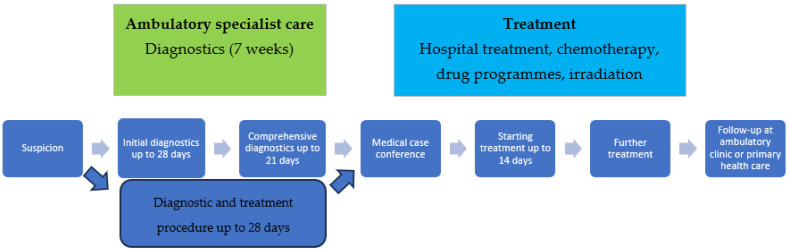
Treatment process containing diagnosis and treatment times resulting from the assumptions of the oncological package.

Figure 2 shows the treatment process, which includes the diagnosis and treatment times resulting from the above-mentioned assumptions of the oncological package.

### 3.2. Chosen Indicators/Analytical Measures for Patients with a Diagnosis of C61

The first step of the analysis of statistical data was establishing the most important indicators/analytical measures for patients with a diagnosis of C61: value of services, number of patients, number of appointments, length of stay, appointment waiting time, and number of appointments per day (Table 1).

The selection of these indicators was based on the availability of reliable data from a verified medical statistics database of the hospital for the period 2018–2022. At the same time, all calculated indicators are widely used for the analysis of patient treatment management effectiveness in medical entities, which enables comparisons between various ways of organizing healthcare systems in different countries. Data on recorded medical events stored in HIS systems are an enormous knowledge resource that can be used to improve processes, medical personnel’s work efficiency, and patient treatment quality. State-of-the-art methods of analysis and visualization of large datasets used in this publication are commonly used in industry as an essential element of Industry 4.0 implementation methodology [25]. The multidimensional database, defined for the purposes of this study, allows a detailed analysis of individual medical events and the grouping of defined indicators at any level of aggregation (Table 2). In particular, the established indicators, i.e., the average length of stay and the average patient wait time, are similar in their method of calculation to the MTTR and MTBF [26] indicators known from TPM [27].

The annual increase in the value of sales can be observed in the list, especially in 2022. The number of appointments of oncological patients with a diagnosis of C61 remained stable, except for a significant decrease in 2020. The average number of appointments/patients during the year was approximately four, and the average length of stay was approximatly 2.9 days. The average appointment waiting time reached a maximum of 46 days in 2020, and in subsequent years, it remained at 43 days. The average number of C61 patient appointments to the hospital per working day was the lowest in 2020, at 44, and in the remaining years, it was 47–50 (Table 2).

Further analysis using the data model was intended to separate the steps of diagnosis and treatment, which enables a detailed analysis of the waiting times and treatment times for selected patients by comparing them to the pattern developed on the basis of existing legal acts/guidelines of scientific associations (Table 3).

On average, on each working day, about 48 appointments of patients with a prostate cancer diagnosis occur in the hospital. Of this, more than 29 appointments are associated with monitoring before and after treatment, 8–9 patients begin radical treatment, and 1–2 multidisciplinary case conferences are held.

The patients’ waiting time for the appointment is, on average, 43 days. Patients wait for the longest for monitoring, 56 days, and the shortest waiting times are for case conferences, 14 days.

Appointments related to initial and comprehensive diagnostics and monitoring have a length of stay of 1 day. Oncological case conferences usually last 1 day, but for hospitalized patients, they may even last a few days (5–6). The average duration of treatment is approximately 11 days, except for radiotherapy, where it is more than 40 days.

The average value of a single appointment in the period analyzed was EUR 337, but there was large variation: EUR 1459 for treatment and less than EUR 20/appointment for initial diagnosis and follow-up (note: partial flat-rate financing is available).

The average treatment cost for one patient was EUR 3066; it was highest for the treatment phase at EUR 5531 and comprehensive diagnostics at EUR 874.

### 3.3. Cost-Intensity of Steps

**Figure 3:**
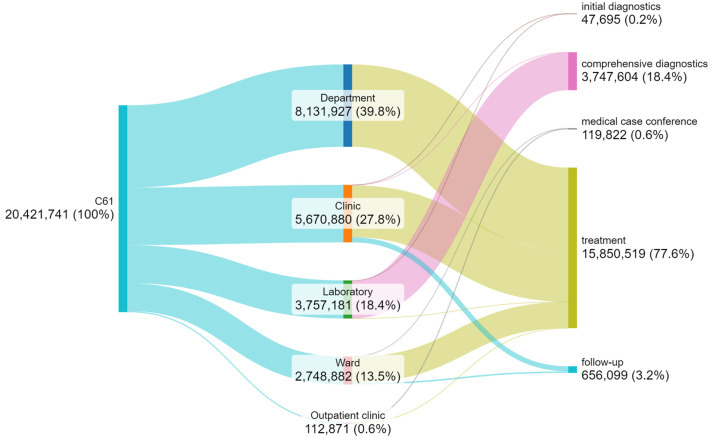
Flow diagram of the value of services (EUR) for patients with C61 in the period 2018–2022 (own work based on hospital data).

The value of services provided in the period of 5 years analyzed (Figure 3) was highest at the treatment step (78%), and together with comprehensive diagnostics (18%), they accounted for 96% of the hospital’s income for this group of patients. This is justified by the highly cost-intensive nature of the treatment methods used and of prostate cancer diagnostics [28,29,30].

The highest value of services was provided by the radiotherapy and brachytherapy departments at EUR 8.1 million.

The total value of services provided in the years 2018–2022 for patients with prostate cancer amounted to EUR 20.4 million.

It should be noted that flat-rate methods of accounting and financing certain medical services may lead to an incorrect allocation of funds in the diagram below.

### 3.4. Number of Patients by Treatment Method

**Figure 4:**
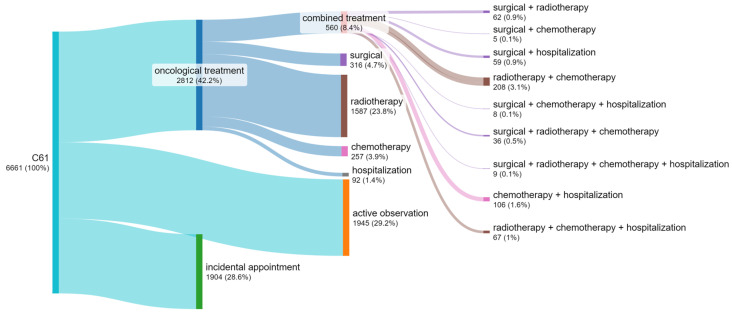
Flow diagram for the number of patients with C61 in the period 2018–2022 (own work based on hospital data).

Of the 6661 oncological patients registered in the hospital with a diagnosis of prostate cancer in 2018–2022 (Figure 4), more than half, i.e., 3849 (58%), were not treated with any of the radical oncological methods. An incidental appointment includes patients with 1–2 diagnostic or consultation appointments, none of which were radical treatment. Active observation includes at least three diagnostic or consultation appointments without the use of radical treatment for the patient.

The largest group of patients underwent treatment with radiotherapy only, comprising 1587 people (56.4% of all patients receiving oncological treatment).

Combination therapy, which involves 11 combinations of different treatment methods, was used in 560 patients (19.9% of all patients receiving oncological treatment).

The relatively low popularity of chemotherapy as an independent method of treatment deserves attention, as chemotherapy frequently occurs in combination with another method of radical treatment of prostate cancer.

### 3.5. Number of Appointments at Individual Steps

**Figure 5:**
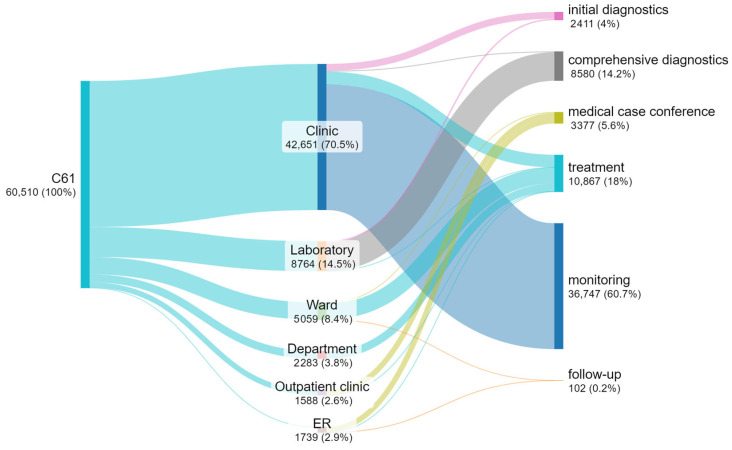
Flow diagram for the number of appointments of patients with C61 in the period 2018–2022 (own work based on hospital data).

The total number of appointments for patients with a diagnosis of C61 at the hospital over 5 years was 60,510 (Figure 5). The largest stream of appointments of patients comes from the clinic (70%) to monitoring (61%).

In laboratories, comprehensive diagnostic appointments (most often imaging diagnostics) are most frequently provided. Most appointments for treated patients occurred in wards (8.4% inpatient treatment), clinics (6.1% outpatient treatment), and specialized departments (3.8% radiotherapeutic treatment). Oncological case conferences were most often held within the outpatient clinic and incidentally as part of inpatient treatment in a hospital ward.

### 3.6. Waiting Time for an Appointment

One of the important parameters of the diagnostic and therapeutic process, which affects the quality of treatment, is the patient’s waiting time for an appointment in days (Table 4). Due to the limitations of the availability of data (there was no reliable information in the system about the actual time of the patient’s registration in the waiting queue), the definition of the waiting time was adopted as the number of days between the patient’s subsequent appointments. This is justified by the registration of reliable information in the system on the start and end date of every appointment. It is, therefore, possible to calculate in a simple way the length of the patient’s stay during each hospital visit and the average time between subsequent appointments in days. Such a method of calculation causes a problem in calculating the waiting time for the patient’s first appointment and longer interruptions in the continuation of treatment for reasons attributable to the patient. After obtaining additional information from the hospital’s visit registration department, it was assumed that the average patient’s waiting time for the first appointment with a diagnosis of prostate cancer was 10 days. For calculation purposes in this data model, an assumption was also made: the reset of the patient waiting counter on 1st of January of every calendar year. Thus, patients who had a break in their treatment of over 12 months do not inflate the calculated average appointment waiting time.

### 3.7. Identification of the Number of Patients Who Do Not Meet the Required Waiting Time Criteria When Treated by Different Methods

**Figure 6:**
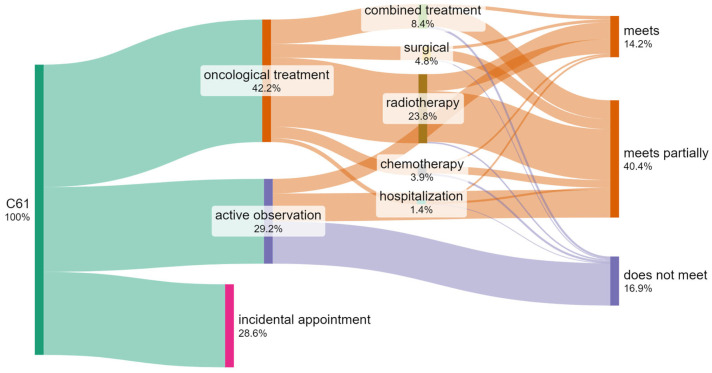
Flow diagram and comparison with the standard for the number of patients with C61 in the period 2018–2022 (own work based on hospital data).

The procedure under the fast-track cancer treatment pathway involves the treatment of the patient within time limits, following the defined steps of diagnosis and treatment of the patient. In principle, therefore, the same patient should appear both at the cancer diagnosis step, the treatment plan establishing step (medical case conference), and the treatment step (no matter the method of treatment). In effect, they pass through the entire pathway, with different effects at each step. It is, therefore, possible that either all steps will be performed within time limits strictly defined by legal provisions (the term “meets”), only some of the steps will be performed in accordance with the time criteria (“meets partially”), or at no step will the time limit be observed (“does not meet”). The diagram below (Figure 6) presents cumulative information on the number of patients who are treated by different methods in the hospital and meet the established waiting time criteria.

For each patient, the appointment waiting time (in days) was calculated, and a comparative test was carried out using the above-defined standard. If the patient’s waiting times for each step of diagnosis and treatment were lower or equal to the number of days specified in the standard, the patient’s treatment met the criteria. If the waiting times during at least one step were higher than the standard, the patient partially met the criteria. If the waiting times during all steps were higher than the standard, the patient did not meet the criteria. It should be noted that only 14.2% of oncological patients with a prostate cancer diagnosis were treated according to the current standard of treatment.

**Figure 7:**
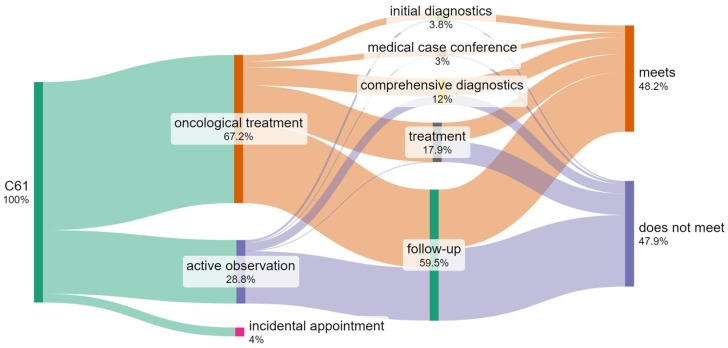
Flow diagram and comparison with the standard for the number of visits of patients with C61 in the period 2018–2022 (own work based on hospital data).

Individual visits within the various steps of the pathway were also analyzed, instead of unique numbers of patients included in diagnostics and oncological treatment; i.e., it was examined what scale of medical services met the given time criteria at each of the steps. In this case, the results were presented using only the following variable: if they meet or do not meet the time criterion (Figure 7).

When carrying out the analyses above, it was demonstrated that some patients appeared in a given unit occasionally (incidentally), which may indicate that the diagnosis and treatment of such a patient occurred at several different independent entities since in no way can we force the patient to limit himself to choosing only one facility (Table 5).

Some of the patients also performed certain procedures at private entities (commercially) and only appeared at a state institution (the hospital where we have conducted our research) for selected (expensive) benefits. In the case of such patients, it is in no way possible to conduct a comparative analysis and evaluate time criteria. Such positions would negatively impact the results of our research, skewing them.

For the reasons specified above, in the last part of the analysis, incidental appointments and monitoring were excluded (Table 6) in order to eliminate the impact of these groups of appointments on the final results of the test and comparison with the standard.

## 4. Discussion

Symptoms of prostate cancer may often be confused with urinary tract diseases, which leads to delays in diagnosis. Time plays a key role in the effectiveness of treatment since the sooner the disease is detected, the better the prognosis [31]. Experts emphasize that early stages of prostate cancer are relatively easy to treat. The treatment of this type of cancer depends on many factors and includes surgery (also using robot technology, such as the da Vinci robot), radiotherapy, and hormone therapy.

It is recommended that men, after 40 years of age, regularly undergo urological examinations. The most common form of prostate cancer is glandular cancer. In developed countries, the number of cases is increasing thanks to testing of PSA (prostate-specific antigen) levels, which allows early detection of the disease [32]. In Poland, prostate cancer incidence has doubled in the last ten years [10].

The key element of prostate cancer diagnosis is the determination of the PSA level in serum. Although this marker is not specific only for cancer, it may also be increased in the case of mild hyperplasia or inflammation of the prostate. Standard PSA levels vary depending on age and are established as follows: 2.5 ng/mL until 49 years of age, 3.7 ng/mL until 54 years of age, 4.0 ng/mL until 59 years of age, 5.4 ng/mL until 64 years of age, and 6.6 ng/mL until 74 years of age. However, there are controversies concerning the effectiveness of PSA-based screening in reducing mortality.

In order to confirm a diagnosis, it is necessary to examine the histopathological material obtained most frequently during a prostate biopsy. A digital rectal examination (DRE) and transrectal ultrasound (TRUS) are also used, although their effectiveness depends on the experience of the physician performing them [33]. In advanced cases, additional imaging studies, such as computer tomography, bone scans, or positron emission tomography (PET), are recommended [34,35].

Various methods are used to treat advanced prostate cancer, including chemotherapy, radiotherapy, hormonal medications, and molecular-targeted therapies. Due to the enormous progress that has been made in uro-oncology, prostate cancer patients have been provided with many new treatment options. Current solutions include both drug therapies and state-of-the-art medical devices. In March 2022, the Ministry of Health decided to provide three molecules in the drug programme for non-metastatic prostate cancer patients: apalutamide, darolutamide, and enzalutamide [36]. Another change introduced in March 2023 was the comprehensive inclusion of all treatment options within one drug programme, which resulted in a change in its name to “B.56. Treatment of patients with prostate cancer (ICD-10: C61)” [37]. Another change includes the inclusion of therapeutic options already reimbursed in the existing programme, as well as the addition of new therapeutic possibilities and indications (apalutamide, cabazitaxel, olaparib, and abiraterone acetate) for patients with advanced prostate cancer.

Modern technologies, such as the da Vinci or CyberKnife systems, increase the precision of surgical procedures and radiotherapy, which leads to better treatment results and lower side effects. In Poland, radical prostatectomy treatments using a robot have been reimbursed by the National Health Fund since April 2022.

The main method of treating prostate cancer patients is radiotherapy combined with oncological surgery [38,39]. Irradiation treatment (teleradiotherapy and brachytherapy of prostate cancer) is used in patients with prostate cancer with staging of cT1-T3 N0 M0 and, in selected cases, in patients with T4 and N(+). At the same time, it is the most cost-intensive treatment method, which, by generating the largest income for the facility, places the greatest burden on the healthcare system.

The diversity of diagnosis and treatment methods for patients with prostate cancer shows how often this process is complicated. Fulfilling all the guidelines and recommendations of scientific associations is often very difficult, and healthcare units repeatedly use different techniques and treatments for the same patient. This is sometimes a result of the facilities’ location, their level of comprehensive care, and their resources. Available publications [40] show, for example, significant geographical variation in the availability of basic treatments, the size of the target population, and coverage areas served by specialized centers, as well as in the provision of additional procedures and services. There is also evidence of increased centralization of basic surgical procedures and regional inequalities in the availability of innovative treatment techniques. According to the study’s authors, proper organization of oncological services allows for a better understanding of the structure, processes, and results of oncological care itself [41]. This is necessary to assess and improve the quality of the provision of oncological care services [42]. It also reduces the time for the diagnosis of the patient and the speed of the provided therapy, which may directly translate into a better clinical effect and improved quality of life for patients [43,44].

## 5. Conclusions

On the basis of the analyses and considerations carried out in the study, the following conclusions were drawn:

1. The time limits for diagnosing and commencing the treatment of patients with diagnosed prostate cancer specified by legal regulations and by guidelines of scientific associations are not observed in 42% of cases.
2. The greatest delays concern the initiation of the treatment (53%) and comprehensive diagnostics (37%).
3. Diagnostic pathways should be modified to facilitate early and rapid detection of prostate cancer and to allow further therapy within the time limit strictly defined by regulations and guidelines of scientific associations. The results presented herein clearly indicate that there is much to be done in terms of the timeliness of the actions taken. Many patients exceed the times established for initial diagnostics, comprehensive diagnostics, or the initiation of treatment (according to DILO).
4. Analysis of the collected data according to the cost criterion indicated that the most cost-intensive methods of treatment are, in order of priority, radiotherapy (irradiation), chemotherapy, and then active observation with laboratory and imaging diagnostics. These areas should be the main areas of interest and subject to control in terms of the regularity of proceedings and implemented activities.
5. The KSO system should also evolve to incorporate the assessment of clinically significant cancer risk, as well as aspects such as precise qualification for biopsy and active surveillance strategies. It is necessary to continue the work initiated by this study by including key issues, in the opinion of the authors, concerning state-of-the-art methods of management of the patient’s treatment processes (and in particular Lean Healthcare 4.0), with the use of the value stream model (VSM) from the perspective of the oncological patient as an example of visual management which can be applied in hospital practice and an indication of the potential of improvement in treatment processes using tools and methods of Lean Healthcare 4.0.

Lean Healthcare 4.0 provides a chance to achieve a competitive advantage for the medical entity, which will implement it effectively.

State-of-the-art methods of process management in oncological facilities include advanced strategies and technologies used to improve the coordination and personalization of diagnosis, treatment, and rehabilitation of patients, as well as the prevention of the recurrence of cancer. Optimizing the treatment processes in these facilities requires simultaneously taking the following into account:

- Quality and clinical results (outcomes);
- The efficiency of the organizational process;
- The improvement in the use of medical resources;
- The reduction in waste (non-value-added activity), in the sense of the lean management methodology;
- The application of the latest standards of treatment and medical technology (best current practice);
- The optimization of process costs.

Such requirements are implemented in multiple organizational and technological improvement projects in medical entities.

## 6. Limitations

- The described model of care refers to data from a Polish hospital and may not be fully representative of healthcare systems in other countries. It is based on data available in Poland and the realities of the Polish healthcare system. However, similar issues to those observed in Poland also occur in many other countries with limited financial resources.
- This study is based on the analysis of data from a single large oncology center in Poland, which constitutes a limitation in terms of generalizing the results to other healthcare institutions. Differences in the organizational structure, availability of services, staffing, and technological resources, as well as clinical practices between centers, may influence the implementation of the diagnostic and therapeutic pathway and the waiting times for services. It should also be noted that patient profiles may be specific to the studied center, which could impact the obtained results. Therefore, the findings presented in this study should be interpreted within the local context, and further research, involving multiple centers, is necessary to more thoroughly validate the observed associations and draw broader conclusions about the organization of oncological care for patients with prostate cancer.

## Figures and Tables

**Table 1:** Definitions of indicators (own work).

Indicator Name	Description	Definition	Measurement Unit
Value	Value of services provided over a given period, taking into account selection criteria	=[Total Value provided]	EUR
Number of patients	Number of unique patients who received services during a given period	=[Number of separate patient ID values]	Integer
Number of appointments	Number of unique appointments carried out during a given period in the framework of the services provided	=[Number of separate appointment ID values]	Integer
Value/Patient	Average value of services per patient in a given period	=[Total Value provided]/[Number of separate patient ID values]	EUR/patient
Value/appointment	Average value of services per appointment in a given period	=[Total Value provided]/[Number of separate appointment ID values]	EUR/appointment
Number of appointments/patient	Average number of appointments per patient in a given period	=[Number of separate appointment ID values]/[Number of separate patient ID values]	Decimal
Length of stay/appointment	Average length of stay of patients per appointment in a given period. Average number of days during one appointment	=AVERAGE([Appointments_Discharge_Date]-[Appointments_Admission_Date])	Decimal
Waiting time/appointment	Average patient waiting time per appointment in a given period. Average number of days between appointments	=AVERAGE([Appointment_N_Admission_Date_N]-[Appointment_N-1_Discharge_Date])	Decimal
Number of appointments/day	Average number of appointments per working day during a given period, taking into account selection criteria	=[Number of separate appointment ID values]/[Number of working days]	Decimal

**Table 2:** Basic hospital medical statistics data for patients with a diagnosis of C61 for the years 2018–2022 (own work based on hospital data).

Year	Value	Number of Patients	Number of Appointments	Value/ Patient	Value/ Appointment	Number of Appointments/ Patient	Length of Stay/ Appointments	Waiting time/ Appointment	Number of Appointments/Day
2018	3,461,145	2784	12,225	1243	283	4.4	2.8	34.1	48.5
2019	3,947,451	3000	12,748	1316	310	4.2	3.1	44.1	50.8
2020	3,860,859	2705	11,141	1427	347	4.1	3.0	46.5	43.7
2021	4,023,156	2798	11,963	1438	336	4.3	3.0	44.4	47.1
2022	5,129,134	2998	12,433	1711	413	4.1	2.8	44.6	49.3
Total	20,421,745	6661	60,510	3066	337	9.1	2.9	42.7	47.9

**Table 3:** Basic hospital medical statistics data for patients with a diagnosis of C61 divided into steps for the years 2018–2022 (own work based on hospital data).

Step	Value	Number of Patients	Number of Appointments	Value/ Patient	Value/ Appointment	Number of Appointments/ Patient	Length of Stay/ Appointments	Waiting time/Appointment	Number of Appointments/Day
Initial diagnosis	47,696	2180	2411	22	20	1.1	1.0	19.6	1.9
Comprehensive diagnostics	3,747,604	4287	8580	874	437	2.0	1.0	27.7	6.8
Medical case conference	119,823	1801	1803	67	66	1.0	1.6	14.0	1.4
Treatment	15,850,522	2866	10,867	5531	1459	3.8	11.4	18.3	8.6
Follow-up	656,100	5321	36,849	123	18	6.9	1.0	56.3	29.2
Total	20,421,745	6661	60,510	3066	337	9.1	2.9	42.7	47.9

**Table 4:** Average appointment waiting time (in days) for patients with a diagnosis of C61 during individual steps for the years 2018–2022 (own work based on hospital data).

Step	2018	2019	2020	2021	2022	Total
Initial diagnosis	12.8	17.6	21.3	21.0	24.4	19.6
Comprehensive diagnostics	19.1	26.9	33.5	28.5	31.5	27.7
Medical case conference	9.9	14.7	14.3	15.7	15.0	14.0
Treatment	18.6	18.4	17.9	18.0	18.7	18.3
Follow-up	44.1	58.4	60.8	59.3	59.5	56.3
Total	34.1	44.1	46.5	44.4	44.6	42.7

**Table 5:** Comparison to the standards for the number of appointments of patients with C61 at individual steps in the period 2018–2022 (own work based on hospital data).

Step	Meet	%	Do Not Meet	%	Total
Initial diagnosis	1951	86%	321	14%	2272
Comprehensive diagnostics	4569	63%	2678	37%	7247
Medical case conference	1269	71%	520	29%	1789
Treatment	5050	47%	5799	53%	10,849
Follow-up	16,309	45%	19,640	55%	35,949
Incidental appointment	2144	89%	260	11%	2404
Total	31,292	52%	29,218	48%	60,510

**Table 6:** Comparison to the standard number of appointments of patients with C61 at diagnosis and treatment steps in the period 2018–2022 (own work based on hospital data).

Step	Meet	%	Do Not Meet	%	Total
Initial diagnosis	1951	86%	321	14%	2272
Comprehensive diagnostics	4569	63%	2678	37%	7247
Medical case conference	1269	71%	520	29%	1789
Treatment	5050	47%	5799	53%	10,849
Total	12,839	58%	9318	42%	22,157

## Data Availability

No new data were created during preparation of this manuscript.

## Abbreviations

The following abbreviations are used in this manuscript:

KSO	National Oncological Network
C61	Code for the diagnosis of prostate cancer in the International Classification of Diseases ICD-10
CSV	Comma-Separated Values (text file format)
NFZ	Polish National Health Fund
DILO	Oncological Diagnostics and Treatment Card
PSA	Prostate Specific Antigen
ICD-10	International Classification of Diseases, Tenth Revision
SPS	Services Provision Site
ODBC	Open Database Connectivity
PHC	Primary health care
ASC	Ambulatory Specialist Care
HT	Hospital Treatment
HIS	Hospital Information System
Industry 4.0	Fourth Industrial Revolution
TPM	Total Productive Maintenance
MTTR	Mean Time To Recovery
MTBF	Mean Time Between Failures
DRE	Digital Rectal Exam
TRUS	Transrectal Ultrasound Scan
PET	Positron Emission Tomography
